# Transumbilical single-incision laparoscopic appendectomy with extracorporeal hand-sewn stump closure in adult patients

**DOI:** 10.20407/fmj.2020-009

**Published:** 2020-12-16

**Authors:** Masashi Isetani, Satoshi Arakawa, Zenichi Morise, Norihiko Kawabe, Hidetoshi Nagata, Yukio Asano, Akihiko Horiguchi

**Affiliations:** 1 Department of Gastroenterological Surgery, Fujita Health University Bantane Hospital, Nagoya, Aichi, Japan; 2 Department of Surgery, Fujita Health University, School of Medicine, Okazaki, Aichi, Japan

**Keywords:** Transumbilical, Single incision, Laparoscopic surgery, Appendectomy

## Abstract

**Objectives:**

We evaluated the clinical outcomes of transumbilical single-incision laparoscopic appendectomy with extracorporeal hand-sewn stump closure in adults.

**Methods:**

One-hundred-and-thirty-one consecutive adults with acute appendicitis were treated with the intention of performing transumbilical single-incision laparoscopic appendectomy with extracorporeal hand-sewn stump closure from July 2012 to December 2017. The procedure completion rate and outcomes were examined. To evaluate the risk factors for conversion, the background data were compared between the patients in whom the procedure was completed versus those in whom the procedure was uncompleted.

**Results:**

The procedure was completed in 113 of 131 patients (86.3%). Single-site surgery was completed in 117 patients (89.3%). The median operation time was 79 (range 30–270) minutes and median intraoperative blood loss was 10 (range 0–394) ml. Postoperative complications occurred in 17 patients (13.0%). Postoperative hospital stay was 6 (range 1–27) days. The 18 patients in whom the procedure could not be completed comprised four patients in whom the stapler was used for intraabdominal stump closure, and 14 patients who were converted to multiport laparoscopic surgery or open surgery. Multivariate analysis showed that the independent risk factors for conversion were age, preoperative abscess, and peri-appendiceal fat density. Receiver operating characteristic curve analysis showed that the cutoff value of peri-appendiceal fat density for conversion was –40.51 Hounsfield units.

**Conclusions:**

Transumbilical single-incision laparoscopic appendectomy with extracorporeal hand-sewn stump closure was safe in adults with acute appendicitis. The risk factors for conversion were age ≥60 years, preoperative abscess, and peri-appendiceal fat density ≥–40.51 Hounsfield units.

## Introduction

Appendectomy is one of the most common emergency abdominal surgeries. Although appendectomy was initially performed by open surgery, laparoscopic appendectomy was first reported in 1983,^1^ and is now widely performed. In single-incision laparoscopic surgery, the laparoscopic surgical procedures are carried out through only one small incision, which has the advantages of minimal invasiveness. Several studies have compared the outcomes of conventional multiport and single-incision laparoscopic appendectomy.^2,3^ The transumbilical single-incision laparoscopic approach is considered feasible for appendectomy,^2^ and its application is increasing nowadays. However, the optimal appendix stump closure technique in laparoscopic appendectomy remains unclear.

Several studies have reported favorable outcomes of transumbilical single-incision appendectomy with extracorporeal stump closure in pediatric patients, a similar closure technique used in open appendectomy,^4,5^ and this method is suggested to be cost-effective.^6,7^ In contrast, the appendix stump is commonly closed using an end-loop, stapler, and clips in laparoscopic appendectomy in adult patients.^7–11^ In our experience, in adult patients with mild ileocecal mobilization, the appendix stump can be closed through a transumbilical procedure in the same way as in the open procedure. However, few studies have reported the outcomes of transumbilical single-incision laparoscopic appendectomy with extracorporeal hand-sewn stump closure in adult patients.

The aims of this study were to evaluate the clinical outcomes of transumbilical single-incision laparoscopic appendectomy with extracorporeal hand-sewn stump closure in adult patients, and to analyze the risk factors for conversion.

## Methods

The study protocol was approved by the institutional review board of Fujita Health University (approval no. HM18-507), within which the work was undertaken, and the study conformed to the provisions of the Declaration of Helsinki established in 1995 (as revised in Brazil in 2013). All patients provided written informed consent in accordance with our institutional guidelines.

For all patients who underwent surgery for acute appendicitis, laparoscopy was first introduced with the intention of performing transumbilical single-incision laparoscopic appendectomy with extracorporeal hand-sewn stump closure, even in patients with abscess formation or perforation. The study cohort comprised 131 consecutive adult patients with acute appendicitis who were surgically treated in our hospital from July 2012 to December 2017. The clinical data were retrospectively reviewed from the medical records. Patients with interval appendectomy were excluded. Interval appendectomy was performed in 11 patients during the study period.

The background factors (sex, age, body mass index (BMI), pathological severity of appendicitis, preoperative abscess formation, presence of coprolite, position of the appendix, peri-appendiceal fat density, white blood cell count (WBC), C-reactive protein (CRP) concentration, and preoperative body temperature) and short-term outcomes (operation time, intraoperative blood loss, postoperative complications, and length of hospital stay) of all patients were analyzed. The completion rate of the intended procedure was also examined.

**Figure 1:**
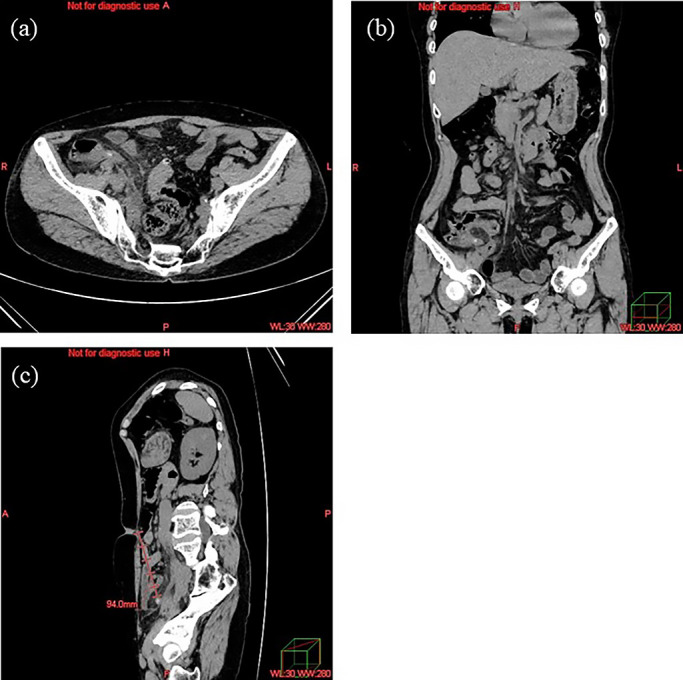
Computed tomography (CT) images at the level of the umbilicus. (a) The appendix is identified, and the peri-appendiceal fat density is measured in Hounsfield units. (b) CT images in the coronal plane. (c) Measuring the distance from the navel to the stump of the appendix on 3D-CT images.

For the patients with conversion from the intended surgery, the conversion styles, rates, and reasons were examined. To evaluate the risk factors for conversion, the patients were classified into the completed group in whom the intended surgery was completed, and the conversion group in whom the intended surgery was not completed. The two groups were compared regarding sex, age, BMI, pathological severity of appendicitis, preoperative abscess formation, presence of coprolite, position of the appendix, peri-appendiceal fat density, distance from the navel to the peritoneum, abdominal wall thickness, distance from the umbilicus to the stump of the appendix (Figure 1), WBC, CRP concentration, and preoperative body temperature.

### Surgical Technique

**Figure 2:**
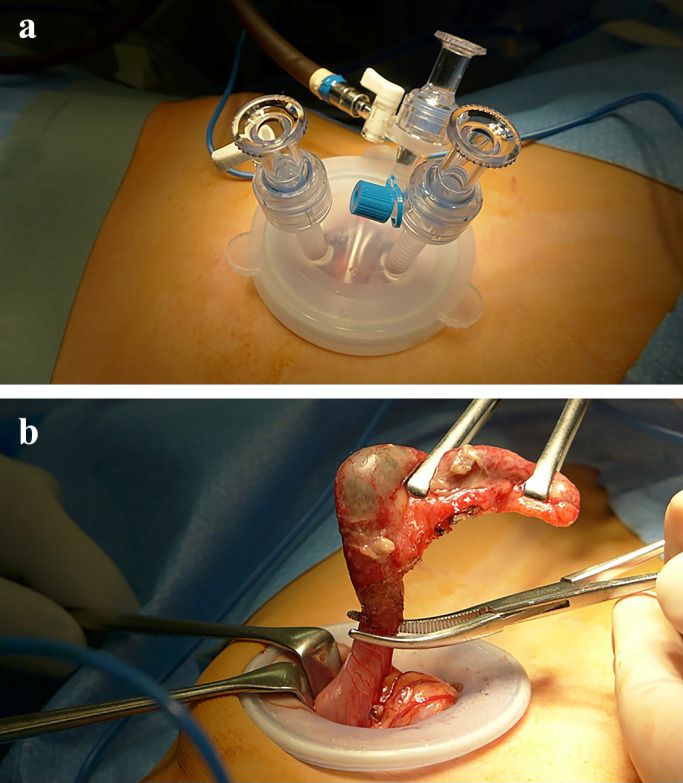
The port arrangement (a) and intraoperative findings (b) of transumbilical single-incision laparoscopic appendectomy with extracorporeal hand-sewn stump closure. A Lap-protector (Hakko, Nagano, Japan) is inserted via a 2–3-cm umbilical incision, and an EZ-access port (Hakko) is attached. Carbon dioxide pneumoperitoneum is created and maintained at 8 mmHg. The operating table is tilted slightly to the left side and with the head down. Two or three 5-mm ports and one 12-mm port are placed using the EZ-access. A 5-mm or 12-mm flexible laparoscope is used. The appendix is grasped and delivered to the extracorporeal space through the umbilical incision, and the pneumoperitoneum is deflated. The appendix and mesoappendix are dissected and ligated. The stump of the appendix is inverted into the base of the cecum wall with a hand sewn purse-string suture in the seromuscular layer of the cecum.

A 2–3-cm umbilical incision was made, a Lap-Protector (Hakko, Nagano, Japan) was inserted, and an EZ-access port (Hakko) was attached (Figure 2A). Carbon dioxide pneumoperitoneum was created, and the pressure was maintained at 8 mmHg. The operating table was tilted slightly to the left side and with the head down. Two or three 5-mm ports and one 12-mm port were placed inside the incision via the EZ-access. A 5-mm or 12-mm flexible laparoscope was used.

The ileocecal segment was mobilized laparoscopically, and the adhesions between the appendix and surrounding tissue were dissected. The appendix was then grasped and removed to the extracorporeal space through the umbilical incision, and the pneumoperitoneum was deflated. The appendix and mesoappendix were dissected and ligated via the same procedure used in open surgery (Figure 2B). The stump of the appendix was inverted into the base of the cecum wall with a hand-sewn purse-string suture in the seromuscular layer of the cecum. The umbilical fascial incision was closed using 3-0 absorbable sutures, and the skin incision was closed using 3-0 absorbable subcuticular sutures.

### Cutoff values of age, BMI, WBC, CRP, body temperature, and peri-appendiceal fat density in predicting the need for conversion

**Figure 3:**
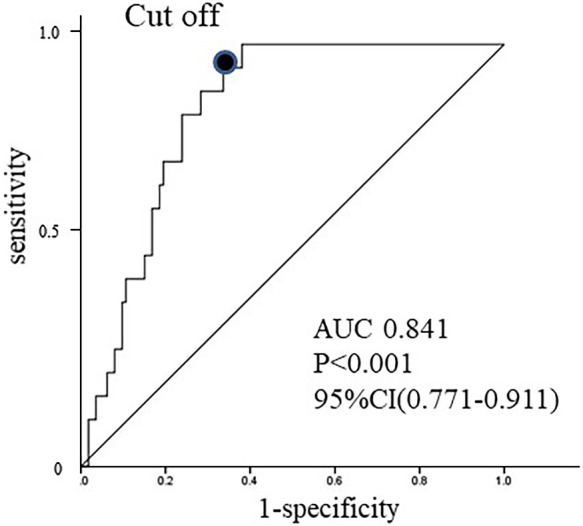
Receiver operating characteristic (ROC) curve for distinguishing the completed group from the conversion group. The area under the curve is 0.841 (95% confidence interval, 0.771–0.911) and the cutoff value for the peri-appendiceal fat density that results in the highest sensitivity and specificity (●) is –40.51 Hounsfield units. Completed group: adult patients in whom the intended procedure was completed; conversion group: adult patients who underwent conversion from the intended procedure.

Receiver operating characteristic curves were plotted using the data for the completed and conversion groups. The area under the curve (AUC) was calculated, and the cutoff value for CRP and peri-appendiceal fat density were set to achieve the highest possible sensitivity and specificity in predicting the need for conversion. When receiver operating characteristic curves were plotted using data for the completed group and the conversion group, the AUC was 0.841 (95% confidence interval, 0.771–0.911) and the cutoff value for peri-appendiceal fat density was set at –40.51 Hounsfield units (HU) (Figure 3); for the CRP concentration, the AUC was 0.766 (95% confidence interval, 0.679–0.853) and the cutoff value was set at 5.99 ng/ml. The cutoff values for age, BMI, WBC, and body temperature were set at ≥60 years, ≥25.0 kg/m^2^, ≥10,000/μl, and ≥38.0°C, respectively.

### Statistical Analysis

Data were expressed as the median (range). Categorical data were expressed as the count number. Pearson’s chi-square test or Fisher’s exact test with Yates correction were used to compare differences in categorical variables, as appropriate. For continuous variables, two-group comparisons were performed using the Mann-Whitney U test. Logistic regression analysis was used for multivariate analysis. SPSS version 19 (SPSS Japan Inc., Tokyo) was used to conduct the statistical analyses. A two-tailed p value of less than 0.05 was considered significant.

## Results

The intended procedure was completed in 113 of 131 patients (86.3%). Single-site surgery was completed in 117 patients (89.3%). The patient characteristics and perioperative data are summarized in Table 1.

The intended procedure could not be completed in 18 patients (conversion group), including four patients in whom the stapler was used for intraabdominal stump closure (S group) and 14 patients who were converted to multiport laparoscopic surgery or open surgery (MO group). In the S group, the reasons for conversion were necrosis or rupture of the appendix in three patients and severe adhesions from previous surgery in one patient. In the MO group, the reasons for conversion were severe adhesion in nine patients (due to previous surgery in three patients), intestinal distension (which led to difficulty in securing a good surgical field) in three patients, and difficulty in localizing the appendix in two patients.

Compared with the completed group, the conversion group had a significantly greater proportion of patients aged ≥60 years, worse severity of appendicitis, greater prevalence of preoperative abscess formation, greater proportion of patients with a peri-appendiceal fat density ≥–40.51 HU, and greater proportion of patients with a CRP concentration ≥5.99 ng/ml (Table 2).

Multivariate analysis showed that the independent risk factors for conversion were age ≥60 years, preoperative abscess formation, and peri-appendiceal fat density ≥–40.51 HU (Table 3).

## Discussion

Transumbilical single-incision laparoscopic appendectomy with extracorporeal hand-sewn stump closure is mainly reported in the treatment of appendicitis in children or adolescents,^12^ as young patients have loose attachment ligaments between the cecum and the retroperitoneum, a short distance between the cecum and umbilicus, and a soft and flexible abdominal wall.^7^ Therefore, the appendix in children is easily introduced to the extracorporeal space through the umbilicus. However, adults have firm attachment ligaments, a firm abdominal wall, and a long distance between the cecum and umbilicus, and so there are only a few reports of transumbilical single-incision laparoscopic appendectomy with extracorporeal hand-sewn stump closure in adult patients.^13^

In the present study, the completion rate of the intended procedure for adult patients was 86.3%, and single-incision surgery was completed in 89.3% of patients. In addition, the overall complication rate in the present study was 13.0%. These results are comparable to the results of previous studies evaluating single-incision surgery for adult appendectomy.^3,14,15^ The conversion rate in the present study of 13.7% (10.7% for conversion from single-site surgery) was acceptable compared with previous studies, and conversions to intracorporeal stump closure, multiport surgery, and open surgery were able to be performed without the additional time and risks associated with usual conversion from single-port surgery to multiport and open surgery. We believe that these results justify our present strategy of first attempting transumbilical single-incision laparoscopic appendectomy with extracorporeal hand-sewn stump closure in adult patients with acute appendicitis.

The advantages of laparoscopic appendectomy compared with open surgery include less pain, a lower wound infection rate, shorter hospital stay, better cosmetic outcome, and earlier return to normal activities; therefore, its application is increasing. However, although single-incision laparoscopic appendectomy is expected to induce less pain, enable a faster recovery, and provide a better cosmetic outcome than conventional laparoscopic surgery, the use of single-incision laparoscopic appendectomy has not markedly increased due to concerns regarding postoperative abscess formation (especially in patients with perforated appendicitis) and high costs.^7^ One of the reasons for the higher cost of single-incision laparoscopic appendectomy compared with conventional laparoscopic surgery is the use of a stapler^16,17^ or other devices^18,19^ for appendix stump closure. An end-loop costs 4,333 yen, and a stapler (Echelon 60) costs 31,000 yen plus 32,000 yen for the cartridge (gold 60). In contrast, silk thread only costs about 150–600 yen. The present study revealed that extracorporeal hand-sewn stump closure without any specific devices is feasible in most adult patients with mild mobilization of the ileocecal area. Thus, the application of extracorporeal hand-sewn stump closure may resolve the issue of the high cost associated with single-incision laparoscopic appendectomy. Furthermore, extracorporeal hand-sewn stump closure may be more accessible for less experienced surgeons during their residency.^13^ In our opinion, the appendectomy procedure described in the present study has both the accessibility of open appendectomy and the advantages of conventional laparoscopic appendectomy, with less expenses.

The two most important complications after appendectomy for severe appendicitis are intra-abdominal abscess formation and surgical site infection,^20–22^ and the incidences of these complications have been evaluated in various settings. Taguchi et al. reported a wound infection rate of 19% for conventional multiport laparoscopic appendectomy for complicated appendicitis,^16^ while Ohno et al. reported a wound infection rate of 7.5% for transumbilical appendectomy for acute, phlegmonous, and perforated appendicitis.^4^ The incidences of intra-abdominal abscess formation (4.6%) and surgical site infection (6.9%) in the present study are comparable to the incidences reported for other procedures.

The reason for the conversion from extracorporeal hand-sewn stump closure in three of four patients was necrosis or rupture of the appendix. In these patients, minimal mobilization of the cecum and appendix was recognized as a risk factor for infection, and so intracorporeal stapler closure was chosen.

The reasons for conversion to multiport or open surgery were severe adhesion in 9 patients (as a result of previous surgery in three patients), intestinal distension (which led to difficulty in securing a good surgical field) in three patients, and difficulty in the localization of the appendix in two patients. Except for the adhesion as a result of previous surgery, these factors are related to the severity of the appendicitis itself. It is reported that appendicitis in older adult patients is often severe due to the decreased lymphoid tissue and blood supply, thin mucosa and an obliterated lumen, wall fibrosis with fatty infiltration, and arteriosclerosis of the small vessels in the appendix.^23^ Furthermore, older adult patients often have comorbidities and do not express typical symptoms.^24^ As older adults tend to have appendicitis in the severe stage, it is reasonable that age is one of the independent risk factors for conversion, irrespective of the peri-appendiceal fat density. The ileocecal mobilization is a minor mobilization outside the ascending colon and at the end of the ileum, and can usually be completed. However, when there is severe adhesion around the ileocecal area with a high risk of mobilization, the stump closure is performed inside the abdominal cavity with a stapler without extracting the appendix. With severe inflammation and paralytic intestinal obstruction, the field of view is narrowed due to distension of the intestines, which leads to a risk of damage to other organs. In such cases, it is necessary to reduce the risk of injury to other organs by tilting the operating table slightly to the left to bring the intestine to the left and creating a working space. However, conversion to laparotomy should be considered when an adequate surgical field cannot be obtained with postural changes.

The present study has some limitations. This was an observational study of patients treated at a single center, and the sample size was small. However, there is likely to have been minimal selection bias, as the present study included all consecutive patients who underwent appendectomy during the study period in our hospital, except for 11 patients who underwent interval appendectomy. Interval appendectomy is not actively applied in our hospital due to the risk of exacerbation of appendicitis (such as abscess formation and perforation) during the period before surgery.

Transumbilical single-incision laparoscopic appendectomy with extracorporeal hand-sewn stump closure has the advantages of relatively low cost and greater accessibility, and was safely applied in adult patients with acute appendicitis. However, the procedure should be applied with caution in older adult patients (≥60 years) and patients with abscess formation or high peri-appendiceal fat density (≥–40.51 HU).

## Figures and Tables

**Table1:** Patient characteristics

Variable	(n=131 consecutive cases)
Sex (male/female)	77/54
Age (years)	38 (10–92)
Body mass index (kg/m^2^)	21.8 (15.2–36.7)
Severity of appendicitis	
• Catarrhal	12
• Phlegmonous	67
• Gangrenous	51
• Perforate	1
Preoperative abscess (+/–)	40/91
Coprolite (+/–)	66/65
Location of the appendix	
• Retrocecal	20
• Pelvic	89
• Subcecal	9
• Pre-ileal	10
• Retro-ileal	3
Preoperative white blood cell count (/μl)	13,300 (3,000–32,600)
Preoperative C-reactive protein (ng/ml)	4.59 (0.01–32.26)
Preoperative body temperature (°C)	37.2 (35.3–40.8)
Operative time (minutes)	79 (30–270)
Blood loss (ml)	10 (0–394)
Drainage tube (+/–)	35/78
Postoperative complications (+/–)	17/114
• Wound infection	9
• Intra-abdominal abscess	6
• Ileus	2
Postoperative length of hospital stay (days)	6 (1–27)

Demographic and perioperative variables of the patients scheduled to undergo transumbilical single-incision laparoscopic appendectomy with extracorporeal hand-sewn stump closure. Data are presented as the number of patients or the median (range).

**Table2:** Univariate analysis of the demographic and preoperative variables to determine the risk factors for conversion

Variable	Completed group (n=113)	Conversion group (n=18)	p
Sex (male/female)	64/49	13/5	0.303
Age (years) (<60/≥60)	98/15	8/10	<0.001
Body mass index (kg/m^2^) (<25.0/≥25.0)	80/15 (18 missing values)	13/4 (one missing value)	0.484
Severity of appendicitis			0.018
• Catarrhal	10	2	
• Phlegmonous	62	5	
• Gangrenous	41	10	
• Perforate	0	1	
Preoperative abscess (+/–)	27/86	13/5	<0.001
Coprolite (+/–)	57/56	9/9	1.000
Appendix location			0.062
• Retrocecal	17	3	
• Pelvic	80	9	
• Subcecal	5	4	
• Pre-ileal	8	2	
• Retro-ileal	3	0	
Peri-appendiceal fat density (Hounsfield units) (<–40.51/≥–40.51)	75/38	1/17	<0.001
Distance from the navel to the peritoneum (mm)	8.0 (3.1–24.5)	8.5 (4.7–19.4)	0.339
Abdominal wall thickness (mm)	25.7 (116–47.5)	26.4 (17.0–47.3)	0.452
Distance from the umbilicus to the stump of the appendix (mm)	97.0 (53.6–148.1)	96.9 (76.1–139.7)	0.547
Preoperative white blood cell count (μl/ml) (<10,000/≥10,000)	33/80	4/14	0.779
Preoperative C-reactive protein concentration (ng/ml) (<5.99/≥5.99)	72/41	2/16	<0.001
Preoperative body temperature (°C) (<38.0/≥38.0)	83/30	14/4	1.000

Completed group: patients who underwent transumbilical single-incision laparoscopic appendectomy with extracorporeal hand-sewn stump closure; conversion group: patients who underwent conversion during transumbilical single-incision laparoscopic appendectomy with extracorporeal hand-sewn stump closure. The distance from the navel to the peritoneum, abdominal wall thickness, and distance from the umbilicus to the stump of the appendix are expressed as the median (range). Categorical data are expressed as the count number. Pearson’s chi-square test or Fisher’s exact test with Yates correction were used to compare differences in categorical variables as appropriate. For continuous variables, two-group comparisons were performed using the Mann-Whitney U test. Patient age, severity of appendicitis, incidence of preoperative abscess formation, peri-appendiceal fat density, and C-reactive protein concentration were significantly higher in the conversion group than the completed group.

**Table3:** Multivariate analysis of the preoperative variables to determine the risk factors for conversion

Variable	Odds ratio	95% confidence interval	p value
Age (years)			
• <60 (n=106, 80.9%)			
• ≥60 (n=25, 19.1%)	5.633	1.615–19.648	0.007
Preoperative abscess			
• No (n=91, 69.5%)			
• Yes (n=40, 30.5%)	3.659	1.010–13.254	0.048
Peri-appendiceal fat density (Hounsfield units)			
• <–40.51 (n=76, 58.0%)			
• ≥–40.51 (n=76, 42.0%)	17.048	2.034–142.885	0.009

Logistic regression analysis was used for the multivariate analysis. Multivariate analysis showed that the independent risk factors for conversion were age ≥60 years, preoperative abscess formation, and peri-appendiceal fat density ≥–40.51 Hounsfield units.
